# Niche, Interspecific Associations, and Community Stability of Dominant Woody Plants in *Betula platyphylla* Forests in the Niyang River Basin, Southeastern Qinghai–Tibet Plateau

**DOI:** 10.3390/plants15121878

**Published:** 2026-06-17

**Authors:** Ngawang Norbu, Hui Zhang, Dorgon Dolma, Rongfang Wang, Zhefei Zeng, Norzin Tso, La Qiong, Junwei Wang

**Affiliations:** 1 Key Laboratory of Biodiversity and Environment on the Qinghai-Tibetan Plateau, Ministry of Education, School of Ecology and Environment, Xizang University, Lhasa 850000, China; anuo046010@163.com (N.N.); 19986738929@163.com (H.Z.); 18208050061@163.com (D.D.); 13228919732@139.com (R.W.); zengzhefei@126.com (Z.Z.); norzin0722@126.com (N.T.); 2 Yani Observation and Research Station for Wetland Ecosystem of the Xizang Autonomous Region, Xizang University, Nyingchi 860000, China

**Keywords:** Niyang River Basin, *Betula platyphylla*, niche, interspecific association, community stability

## Abstract

Niche and interspecific association are important components of community ecology and are of great significance for revealing the mechanisms of community assembly and its stability. In this study, the woody plant communities of *Betula platyphylla* Sukaczev forests in the Niyang River Basin of southeastern Qinghai–Tibet Plateau were taken as the research object. The niche, interspecific association, and community stability of dominant tree species in *B. platyphylla* forests were analyzed using the Levins index (*B*_L_), Shannon index (*B*_S_), Pianka index (*O*_ik_), Schoener index (*C*_ik_), variance ratio (VR), chi-square test, association coefficient (AC), Spearman rank correlation, and M. Godron stability methods. The results showed that a total of 71 woody plant species were recorded across 48 plots, mainly belonging to Rosaceae, Ericaceae, and Caprifoliaceae. *B. platyphylla*, *Quercus aquifolioides* Rehder & E. H. Wilson, *Sorbus rehderiana* Koehne, and *Berberis gyalaica* Ahrendt had relatively large niche breadths, indicating strong resource utilization ability and a wide range of spatial adaptation. They were the main constructive species and dominant species of *B. platyphylla* forest communities in this basin. The overall niche overlap of woody plant communities was relatively low, indicating relatively obvious differentiation in resource utilization among different species. Interspecific association analysis showed that the dominant species in the tree layer exhibited an overall significantly positive association, whereas those in the shrub layer exhibited an overall non-significantly positive association. The associations between species pairs were mainly non-significant, and the overall interspecific association was weak. Most species showed a relatively independent distribution pattern, reflecting weak interspecific competition within the community. Community stability analysis showed that the Euclidean distance between the tree layer and the theoretical stability point (20, 80) was 20.17, whereas that of the shrub layer was 27.98, indicating that the tree layer was more stable than the shrub layer. Overall, the community may not yet have reached a fully stable state. The results provide important references for biodiversity conservation, vegetation restoration, and sustainable forest management in alpine canyon ecosystems. Future studies should incorporate environmental factors such as soil properties and hydrothermal conditions to further reveal the ecological mechanisms driving community succession and stability.

## 1. Introduction

Niche is an important foundation of community ecology research and is of great significance for revealing the distribution patterns, resource utilization modes, and coexistence mechanisms of species in plant communities within multidimensional environmental resource space [1]. A niche refers to the sum of resources a population can utilize in time and space, as well as the interactions it has with other populations [2,3]. Among them, niche breadth represents the extent of the resource utilization range of a species, whereas niche overlap reflects the similarity in resource utilization among different species and the potential intensity of competition [4]. Therefore, niche analysis has become an important approach for understanding community structure, species coexistence, and patterns of community succession [5].

Meanwhile, interspecific association, as the manifestation of associations among species in spatial distribution, is an important indicator for analyzing species interaction relationships within communities [6]. It can infer ecological relationships among species, such as mutual facilitation, competitive exclusion, or relative independence, thereby reflecting the organizational structure and successional process of communities [7,8]. Community stability is the comprehensive attribute by which plant communities maintain relatively stable structure and function when subjected to external disturbance, and is one of the core issues in current plant ecology research [9]. The formation and maintenance of community stability depend on complex interspecific interaction networks, while niche differentiation and interspecific association are two key aspects affecting community stability [10,11]. In general, stronger niche differentiation helps to reduce competition among species for limited resources and promotes species coexistence; whereas a reasonable pattern of interspecific association helps to enhance the coordination of the internal structure of communities and improve the ability of communities to resist disturbance and self-regulate. Therefore, integrating niche, interspecific association, and community stability in research has become an important approach for elucidating community assembly mechanisms and ecological restoration processes [12].

Niche and interspecific association, as important components for revealing community assembly mechanisms and species coexistence relationships, have received extensive academic attention in recent years and have been widely studied in various ecosystems such as forests, deserts, and grasslands. Tu et al. [13], in their study of tree species in the karst hills of Guilin, found that among 231 species pairs, 108 pairs were positively correlated, 115 pairs were negatively correlated, and 8 pairs were unrelated, with positive and negative association rates reaching 93.9%. Most species pairs showed no significant correlation, and this independence may result from the high habitat heterogeneity in this region, which leads to species niche differentiation. In alpine grasslands and desertified vegetation, there are significant differences in species niche breadth, degree of overlap, and interspecific association characteristics under different environmental gradients, reflecting the adaptive strategies of plants to environmental change and the process of community reorganization [3]. In artificial sand-fixation vegetation and restoration communities, researchers further found that community stability is closely related to niche overlap and interspecific association, and that different community types show obvious differences in resource utilization modes, interspecific relationships, and stability levels [9]. In forest ecosystems, relevant studies have also shown that most species pairs within communities often exhibit weak associations or relatively independent distributions, and that niche differentiation plays an important role in reducing competition, maintaining species coexistence, and promoting community succession [8,14].

The Qinghai–Tibet Plateau is one of the regions most sensitive to global climate change [15], and is also a hotspot region for studying ecosystem responses to climate change [16]. The Niyang River Basin is located in the southeastern Qinghai–Tibet Plateau, where its unique hydrothermal conditions have nurtured rich yet fragile forest ecosystems. As a common forest type with important ecological functions in this basin, *Betula platyphylla* Sukaczev forests play important roles in water conservation, soil conservation, maintenance of regional biodiversity, and promotion of secondary community succession. However, studies on understory woody plant communities in *B. platyphylla* forests in the Niyang River Basin remain relatively limited, particularly with a lack of systematic understanding of community assembly mechanisms, interspecific interactions, and community stability.

This study focused on the woody plant communities of *B*. *platyphylla* forests in the Niyang River Basin. The objectives of this study were to (1) characterize the niche breadth and niche overlap of dominant woody plant species; (2) analyze interspecific associations among dominant species; and (3) evaluate community stability based on species composition and population structure. By addressing these objectives, this study aims to improve understanding of the community structure and ecological relationships of *B. platyphylla* forests in the Niyang River Basin and provide a basis for future studies on community assembly and vegetation dynamics in this region.

## 2. Results

### 2.1. Importance Values and Niche Breadth Characteristics of the Main Dominant Species in B. platyphylla Forest Communities

In the *B. platyphylla* forest communities investigated in this study, a total of 71 woody plant species were recorded, belonging to 40 genera and 22 families, mainly including Rosaceae, Ericaceae, Caprifoliaceae, and Salicaceae. These dominant families are widely distributed in high-elevation mountain ecosystems and exhibit strong cold tolerance and environmental adaptability. Their dominance reflects the adaptation of *B*. *platyphylla* forest communities in the study area to high-altitude and low-temperature conditions. Based on the importance values of species, the top 12 tree species and the top 15 shrub species were selected as the main dominant species for analyses of niche breadth, niche overlap values, interspecific association, and community stability. Rare species, due to their low frequency and small importance values, were not included in the analysis of interspecific relationships; however, they still play an important ecological role in maintaining community species diversity and ecosystem functions.

The importance values and niche breadths of the main species in the tree layer and shrub layer of *B. platyphylla* forest communities in the Niyang River Basin are shown in Table 1 and Table 2. In the tree layer, *B. platyphylla* had the highest importance value (27.43%), followed by *Quercus aquifolioides* Rehder & E. H. Wilson and *Salix daltoniana* Andersson, with importance values of 11.42% and 3.24%, respectively, while *Populus mainlingensis* C. Wang & S. L. Tung had the lowest importance value, at 0.30%. In terms of niche breadth, although the rankings of niche breadth of dominant species obtained by the two niche breadth calculation methods differed slightly, the results were generally consistent. The Levins index (*B*_L_) of the main species in the tree layer ranged from 2.00 to 37.16, and the Shannon index (*B*_S_) ranged from 0.69 to 3.71. Among the two indices, *B. platyphylla* had the largest niche breadth (*B*_L_ = 37.16; *B*_S_ = 3.71), followed by *Q. aquifolioides* (*B*_L_ = 16.57; *B*_S_ = 3.00) and *Sorbus rehderiana* Koehne (*B*_L_ = 13.09; *B*_S_ = 2.64), whereas *P. mainlingensis* had the smallest niche breadth (*B*_L_ = 2.00; *B*_S_ = 0.69).

In the shrub layer, *Rhododendron bulu* had the highest importance value (5.18%), followed by *Berberis gyalaica* Ahrendt and *Lonicera tangutica* Maxim., with importance values of 4.02% and 3.53%, respectively, while *Lonicera hispida* Pall. ex Roem. & Schult. had the lowest importance value (0.98%). The Levins index (*B*_L_) of the main species in the shrub layer ranged from 3.20 to 22.50, and the Shannon index (*B*_S_) ranged from 1.35 to 3.26. Among the two indices, *B. gyalaica* had the largest niche breadth (*B*_L_ = 22.50; *B*_S_ = 3.26), followed by *L. tangutica* (*B*_L_ = 17.63; *B*_S_ = 3.14) and *Cotoneaster submultiflorus* Popov (*B*_L_ = 11.82; *B*_S_ = 2.76), whereas *Rhododendron vellereum* Hutch. ex Tagg had the smallest niche breadth (*B*_L_ = 3.20; *B*_S_ = 1.48).

Overall, in the ranking of niche breadth in *B. platyphylla* forest communities, *Q. aquifolioides*, *S. rehderiana*, *B. gyalaica*, and *L. tangutica* ranked among the top, indicating that these species occupy relatively wide niches in *B. platyphylla* forest communities, possess strong environmental adaptability, have strong resource utilization ability, and occupy a dominant position in competition.

### 2.2. Niche Overlap and Similarity Characteristics of Dominant Species

The Pianka index (*O*_ik_) and Schoener index (*C*_ik_) of the main species in the tree layer and shrub layer of *B. platyphylla* forest communities are shown in Table 3 and Table 4.

The results of the Pianka niche overlap analysis showed that, in the tree layer, the niche overlap index (*O*_ik_) of 66 species pairs composed of 12 main dominant species ranged from 0.00 to 0.58, with a mean value of 0.17 (Table 3). There were 4 species pairs with *O*_ik_ ≥ 0.45, accounting for 6% of the total pairs, namely *AG*-*PL* (*O*_ik_ = 0.58), *SR*-*QA* (*O*_ik_ = 0.48), *SR*-*PD* (*O*_ik_ = 0.47), and *SD*-*BP* (*O*_ik_ = 0.46). There were 10 species pairs with 0.3 ≤ *O*_ik_ < 0.45, accounting for 15.15% of the total pairs, such as *CH*-*PD* (*O*_i_ = 0.40), *PM*-*LG* (*O*_ik_ = 0.38), and *PL*-*SR* (*O*_ik_ = 0.38). There were 52 species pairs with *O*_ik_ < 0.3, accounting for 78.79% of the total species pairs, among which 11 pairs had no niche overlap (*O*_ik_ = 0), accounting for 16.66% of the total pairs, such as *LG*-*PR*, *BU*-*PD*, and *CH*-*LG*.

The results of Schoener niche similarity analysis showed that, in the tree layer, the niche similarity index (*C*_ik_) of 66 species pairs composed of 12 main dominant species ranged from 0.00 to 0.43, with a mean value of 0.13 (Table 3). There were 2 species pairs with *C*_ik_ ≥ 0.4, accounting for 3.03% of the total species pairs, namely *PL*-*AG* (*C*_ik_ = 0.43) and *QA*-*SR* (*C*_ik_ = 0.40). There were 10 species pairs with 0.25 ≤ *C*_ik_ < 0.4, accounting for 15.15% of the total species pairs, such as *QA*-*PD*, *PR*-*CH*, *PD*-*SR*, *SR*-*PL*, and *BP*-*PL*. There were 54 species pairs with *C*_ik_ < 0.25, accounting for 81.82% of the total species pairs, among which 11 pairs showed no niche similarity (*C*_ik_ = 0), accounting for 16.66% of the total pairs, such as *PR*-*LG*, *PD*-*BU*, and *LG*-*CH*.

In the shrub layer, the niche overlap index (*O*_ik_) of 105 species pairs composed of 15 main dominant species ranged from 0.00 to 0.73, with a mean value of 0.18 (Table 4). There were 10 species pairs with *O*_ik_ ≥ 0.45, accounting for 9.52% of the total pairs, such as *LH*-*DF* (*O*_ik_ = 0.73), *LF*-*RT* (*O*_ik_ = 0.63), *CS*-*LV* (*O*_ik_ = 0.58), *BG-RM* (*O*_ik_ = 0.57), and *RM*-*RT* (*O*_ik_ = 0.50). There were 16 species pairs with 0.3 ≤ *O*_ik_ < 0.45, accounting for 15.23% of the total pairs, such as *CS*-*RS* (*O*_ik_ = 0.43), *BG*-*LT* (*O*_ik_ = 0.42), *RL*-*RT* (*O*_ik_ = 0.40), and *CA*-*RM* (*O*_ik_ = 0.40). There were 79 species pairs with *O*_ik_ < 0.3, accounting for 75.24% of the total species pairs, among which 10 pairs showed no niche overlap (*O*_ik_ = 0), accounting for 9.52% of the total pairs, such as *LH*-*LF*, *LH-CB*, *LH-RL*, *RL-CB*, *RL-RV*, and *CB-RT*.

In the shrub layer, the niche similarity index (*C*_ik_) of 105 species pairs ranged from 0.00 to 0.56, with a mean value of 0.16 (Table 4). There were 7 species pairs with *C*_ik_ ≥ 0.4, accounting for 6.66% of the total species pairs, namely *DF*-*LH* (*C*_ik_ = 0.56), *LV*-*RT* (*C*_ik_ = 0.50), *RT*-*LF* (*C*_ik_ = 0.48), *RT*-*RM* (*C*_ik_ = 0.47), *RS*-*BG* (*C*_ik_ = 0.43), *CS*-*BG* (*C*_ik_ = 0.40), and *LT*-*BG* (*C*_ik_ = 0.40). There were 21 species pairs with 0.25 ≤ *C*_ik_ < 0.4, accounting for 20.00% of the total species pairs, such as *LV*-*CA* (*C*_ik_ = 0.39), *LV*-*CS* (*C*_ik_ = 0.38), and *RS*-*CS* (*C*_ik_ = 0.38). There were 76 species pairs with *C*_ik_ < 0.25, accounting for 72.38% of the total species pairs, among which 10 pairs showed no niche similarity (*C*_ik_ = 0), accounting for 9.52% of the total pairs, such as *LF-LH*, *CB-LH*, *RL-LH*, *CB-RL*, *RV-RL*, and *RT-CB*.

In summary, the Schoener niche similarity index and Pianka niche overlap index of the vast majority of species pairs in the tree layer and shrub layer of *B. platyphylla* forest communities were low, indicating a high degree of niche differentiation among woody plants in *B. platyphylla* forests. There are large differences among species in resource requirements, with low niche overlap and similarity, and relatively weak interspecific competition pressure.

### 2.3. Interspecific Association Analysis of Dominant Species

#### 2.3.1. Overall Association Analysis of Dominant Species

Based on the presence–absence data matrix of species in 48 quadrats of *B. platyphylla* forest communities, the variance ratio method was used to test the overall association of the main dominant species (Table 5). The results showed that the variance ratio (VR) of the tree layer was 1.854 (VR > 1), indicating that the dominant species were overall positively associated. The test statistic (W) was 89.04. From the table, χ^2^_0.95_ (48) = 65.17 and χ^2^_0.05_ (48) = 33.10; since W > χ^2^_0.95_, it indicates that the overall association of dominant species in the tree layer showed a significantly positive association. In the shrub layer, VR = 1.268 (VR > 1), indicating that the dominant species were overall positively associated. The test statistic (W) was 60.89, which fell within the interval between χ^2^_0.05_ (48) and χ^2^_0.95_ (48), indicating that the overall association of the main dominant species in the shrub layer showed a non-significantly positive association.

#### 2.3.2. χ^2^ Test of Interspecific Association

**Figure 1:**
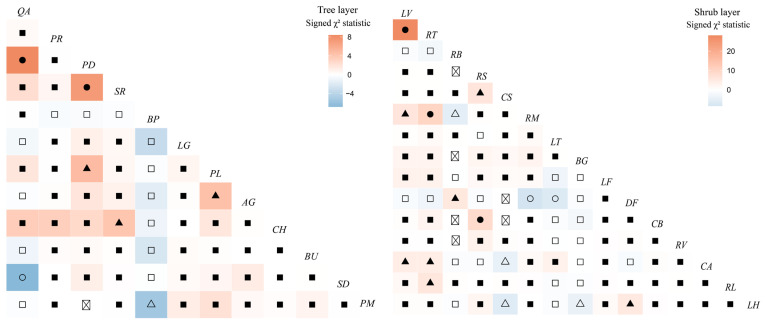
χ^2^ test of the Main Dominant Species in the tree layer and shrub layer of *B. platyphylla* forests. Note: ● Extremely significant positive association, ▲ significant positive association, ■ non-significant positive association, ⊠ no association, ☐ non-significant negative association, △ significant negative association, ○ extremely significant negative association. Positive and negative signed χ^2^ statistics indicate positive and negative interspecific associations, respectively.

The χ^2^ test results of the main dominant species in the tree layer and shrub layer of *B. platyphylla* forest communities are shown in Figure 1. Among the 66 species pairs composed of 12 main dominant species in the tree layer, there were 50 positively associated pairs, 15 negatively associated pairs, and 1 unassociated pair, accounting for 75.75%, 22.72%, and 1.51% of the total species pairs, respectively. Among them, 58 species pairs showed non-significant association, accounting for 87.87% of the total species pairs, and the ratio of positive to negative associations was 3.33. Among the positively associated species pairs, there were 2 pairs with extremely significant positive association, namely *QA*-*PD* and *PD*-*SR*, 3 pairs with significant positive association, and 45 pairs with non-significant positive association, accounting for 3.03%, 4.54%, and 68.18% of the total species pairs, respectively. Among the negatively associated species pairs, there was 1 pair with an extremely significant negative association, 1 pair with a significant negative association, and 13 pairs with a non-significant negative association, accounting for 1.51%, 1.51%, and 19.69% of the total species pairs, respectively.

In the shrub layer, among the 105 species pairs, there were 71 positively associated pairs, 28 negatively associated pairs, and 6 unassociated pairs, accounting for 67.62%, 26.66%, and 5.71% of the total species pairs, respectively. Among them, 83 species pairs showed non-significant association, accounting for 79.05% of the total species pairs, and the ratio of positive to negative associations was 2.54. Among the positively associated species pairs, there were 3 pairs with extremely significant positive association, namely *LV*-*RT*, *RT*-*RM*, and *RS*-*CB*, 7 pairs with significant positive association, and 61 pairs with non-significant positive association, accounting for 2.86%, 6.66%, and 58.10% of the total species pairs, respectively. Among the negatively associated species pairs, there were 2 pairs with extremely significant negative association, 4 pairs with significant negative association, and 22 pairs with non-significant negative association, accounting for 1.90%, 3.80%, and 20.96% of the total species pairs, respectively.

#### 2.3.3. AC Association Coefficient Analysis of Dominant Species

**Figure 2:**
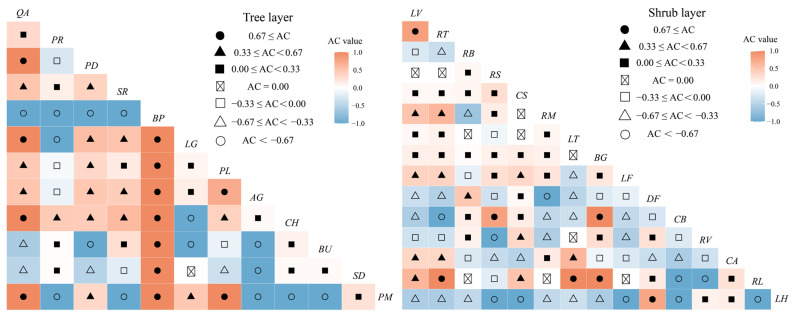
AC Association Coefficient of the Main Dominant Species in the tree layer and shrub layer of *B. platyphylla* forests. Note: ● strong positive association, ▲ moderate positive association, ■ weak positive association, ⊠ no association, ☐ weak negative association, △ moderate negative association, ○ strong negative association.

The results of the AC association analysis of the main dominant species in the tree layer and shrub layer of *B. platyphylla* forest communities are shown in Figure 2. Among the 66 species pairs in the tree layer, there were 41 positively associated pairs, 24 negatively associated pairs, and 1 unassociated pair, accounting for 62.12%, 36.36%, and 1.51% of the total species pairs, respectively. Among them, 18 species pairs showed weak association, accounting for 27.27% of the total species pairs, and the ratio of positive to negative associations was 1.71. Among the positively associated species pairs, there were 13 pairs with strong positive association, 15 pairs with moderate positive association, and 13 pairs with weak positive association, accounting for 20%, 22.72%, and 20% of the total species pairs, respectively. Among the negatively associated species pairs, there were 15 pairs with strong negative association, 4 pairs with moderate negative association, and 5 pairs with weak negative association, accounting for 22.72%, 6.06%, and 7.57% of the total species pairs, respectively.

In the shrub layer, among the 105 species pairs, there were 48 positively associated pairs, 47 negatively associated pairs, and 10 unassociated pairs, accounting for 45.71%, 44.76%, and 9.52% of the total species pairs, respectively. Among them, 44 species pairs showed weak association, accounting for 41.90% of the total species pairs, and the ratio of positive to negative associations was 1.02. Among the positively associated species pairs, there were 7 pairs with strong positive association, 12 pairs with moderate positive association, and 29 pairs with weak positive association, accounting for 6.66%, 11.43%, and 27.62% of the total species pairs, respectively. Among the negatively associated species pairs, there were 10 pairs with strong negative association, 22 pairs with moderate negative association, and 15 pairs with weak negative association, accounting for 9.52%, 20.95%, and 14.29% of the total species pairs, respectively.

#### 2.3.4. Spearman Rank Correlation Test of Dominant Species

**Figure 3:**
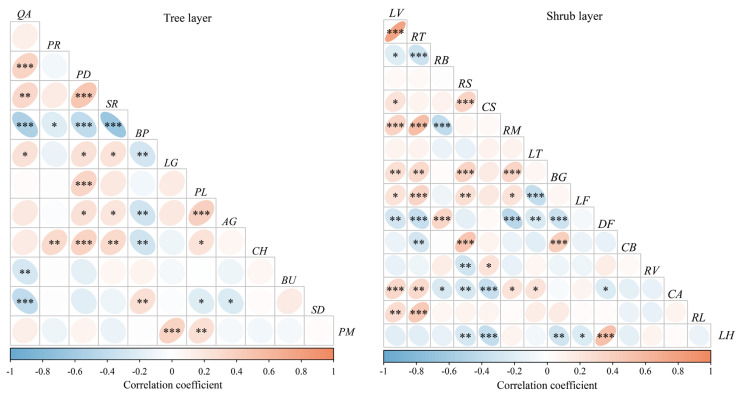
Spearman Rank Correlation Test of the Main Dominant Species in the tree layer and shrub layer of *B. platyphylla* forests. Note: * *p* < 0.05, ** *p* < 0.01, and *** *p* < 0.001.

The results of the Spearman rank correlation test of the main dominant species in the tree layer and shrub layer of *B. platyphylla* forest communities are shown in Figure 3. Among the 66 species pairs in the tree layer, there were 37 positively associated pairs, 28 negatively associated pairs, and 1 unassociated pair, accounting for 56.06%, 42.42%, and 1.51% of the total species pairs, respectively. Among them, 37 species pairs showed non-significant association, accounting for 56.06% of the total species pairs, and the ratio of positive to negative associations was 1.32. Among the positively associated species pairs, there were 11 pairs with extremely significant positive association, 6 pairs with significant positive association, and 20 pairs with non-significant positive association, accounting for 16.66%, 9.10%, and 30.30% of the total species pairs, respectively. Among the negatively associated species pairs, there were 8 pairs with extremely significant negative association, 3 pairs with significant negative association, and 17 pairs with non-significant negative association, accounting for 12.12%, 4.54%, and 25.76% of the total species pairs, respectively.

In the shrub layer, among the 105 species pairs, there were 60 positively associated pairs and 45 negatively associated pairs, accounting for 57.14% and 42.85% of the total species pairs, respectively. Among them, 62 species pairs showed non-significant association, accounting for 59.04% of the total species pairs, and the ratio of positive to negative associations was 1.33. Among the positively associated species pairs, there were 18 pairs with extremely significant positive association, 6 pairs with significant positive association, and 36 pairs with non-significant positive association, accounting for 17.14%, 5.71%, and 34.28% of the total species pairs, respectively. Among the negatively associated species pairs, there were 15 pairs with extremely significant negative association, 4 pairs with significant negative association, and 26 pairs with non-significant negative association, accounting for 14.28%, 3.80%, and 24.76% of the total species pairs, respectively.

### 2.4. Community Stability Analysis of B. platyphylla Forest Communities

**Figure 4:**
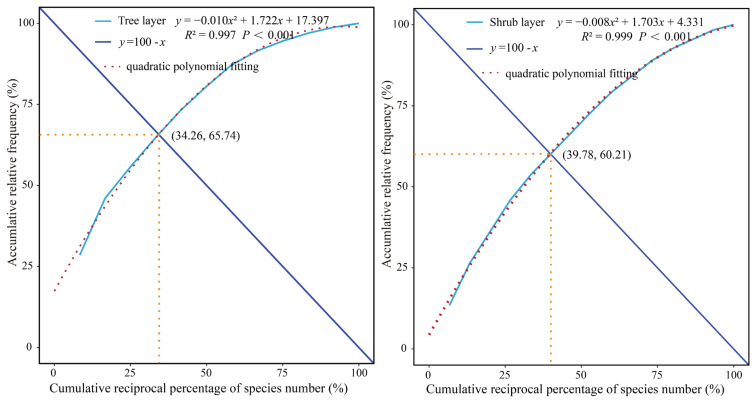
Community Stability Analysis of the tree layer and shrub layer of *B. platyphylla* Forests. Note: The theoretical stability point (20, 80) represents the ideal stable state of the community. Community stability was evaluated using the Euclidean distance between the observed intersection point of the fitted curve and the stability model (y = 100 − x) and the theoretical stability point. Smaller Euclidean distances indicate higher community stability.

To further verify the community stability of the tree layer and shrub layer of *B. platyphylla* forests, the M.Godron function stability model was constructed (Figure 4). The stability model y = 100 − x and the nonlinear fitting equation between the cumulative reciprocal of total species number and cumulative relative frequency in the tree layer is y = −0.010x^2^ + 1.722x + 17.397, with a coefficient of determination (*R*^2^) of 0.997 and *p* < 0.01, intersecting at (34.26, 65.74). The Euclidean distance from the theoretical stability point (20, 80) is 20.17. The relatively large deviation of its actual dynamic trajectory from the ideal stable state suggests that the tree-layer community may not yet represent a highly stable state.

The stability model y = 100 − x and the nonlinear fitting equation between the cumulative reciprocal of total species number and cumulative relative frequency in the shrub layer is y = −0.008x^2^ + 1.703x + 4.331, with a coefficient of determination (*R*^2^) of 0.999 and *p* < 0.01, intersecting at (39.78, 60.21). The Euclidean distance from the theoretical stability point (20, 80) is 27.98. The deviation of the observed dynamic trajectory from the theoretical stable state is relatively high, suggesting that the shrub-layer community may not have reached a fully stable condition.

## 3. Discussion

### 3.1. Niche Breadth and Overlap Analysis of Dominant Species in the Community

The degree of niche overlap is an important indicator for evaluating the intensity of interspecific resource competition [17], reflecting the similarity of species in ecological characteristics and resource utilization [18]. The higher the degree of niche overlap, the more intense the competition among species; conversely, it indicates that species achieve optimal resource allocation through niche differentiation. In this study, the Pianka niche overlap index (*O*_ik_) and Schoener similarity index (*C*_ik_) of the main dominant species were generally low. The mean values in the tree layer were 0.17 and 0.13, respectively, with 78.79% of species pairs having *O*_ik_ < 0.3 and 81.82% having *C*_ik_ < 0.25. In the shrub layer, the mean values of the Pianka niche overlap index (*O*_ik_) and Schoener similarity index (*C*_ik_) were 0.18 and 0.16, respectively, with 75.24% of species pairs having *O*_ik_ < 0.3 and 72.38% having *C*_ik_ < 0.25. This indicates that most species in *B. platyphylla* forest plant communities differ considerably in ecological and biological characteristics and show some differentiation in the use and demand of environmental resources, which may reflect relatively weak interspecific competition, consistent with the findings of Li et al. [19]. It should be noted, however, that low niche overlap does not necessarily indicate weak interspecific competition alone; such patterns may also result from spatial segregation along elevation gradients, environmental filtering, dispersal limitation, or differences in species abundance.

In the tree layer, the two groups of dominant species with the highest niche overlap and similarity values were *Picea likiangensis* var. *linzhiensis* W. C. Cheng & L. K. Fu (*PL*)-*Abies georgei* Orr (*AG*) and *Q. aquifolioides* (*QA*)-*S. rehderiana* (*SR*). In the shrub layer, they were *L. hispida* (*LH*)-*Dasiphora fruticosa* (L.) Rydb. (*DF*) and *Rhododendron triflorum* Hook. (*RT*)-*Leptodermis forrestii* Diels (*LF*), indicating that these species pairs have convergent ecological strategies in the dimension of resource utilization, and exhibit high consistency in the spatial allocation and utilization patterns of environmental resources.

Some studies have shown that species with relatively wide niches usually have a higher degree of overlap in resource utilization dimensions, thereby leading to increased niche overlap and similarity [20]. In the tree layer of this study, *Q. aquifolioides* (*QA*)-*S. rehderiana* (*SR*), and in the shrub layer, *B. gyalaica* (*BG*) with *L. tangutica* (*LT*) and *C. submultiflorus* (*CS*), exhibited relatively high niche overlap and niche similarity, indicating that these species have strong consistency in resource utilization patterns and ecological requirements. However, in the tree layer, the two species with the largest niche breadths, *B. platyphylla* (*BP*) and *Q. aquifolioides* (*QA*), did not show prominent niche overlap and niche similarity, indicating that there is no fixed relationship between niche breadth and niche overlap [21].

### 3.2. Interspecific Association of Dominant Species and Community Stability Analysis

Interspecific association characteristics can effectively reveal the developmental trend and stability of community succession [22]. The results of this study showed that the overall association of the tree layer in *B. platyphylla* forests in the Niyang River Basin was a significantly positive association, indicating that the coexistence relationships among species in the tree layer were relatively close, possibly affected by consistent environmental filtering or facilitation, such as soil moisture, nutrients, and light environment, thereby leading to consistent spatial distribution of species. In contrast, although the overall association of the shrub layer was positive, it was not significant, indicating that the relationships among species in the shrub layer were relatively weak and may be more strongly affected by microenvironmental heterogeneity, regeneration dynamics, or disturbance factors, resulting in greater randomness in their distribution. This is similar to the results of Wei et al. [23] on woody plant communities of Michelia shiluensis forests in Diaoluo Mountain and Ai et al. [18] on plant communities of elfin forests in Mulinzi.

The association characteristics of interspecific relationships provide a key basis for analyzing species coexistence mechanisms and differences in habitat requirements among different species [5]. Combined with the χ^2^ test, AC association, and Spearman rank correlation test analyses in this study, the results showed that the number of positively associated species pairs among the main dominant species in the tree layer and shrub layer was greater than that of negatively associated species pairs, and the associations of most species pairs did not reach a significant level. This indicates that the overall interactions among species within the community were relatively weak. The few positively associated species pairs that reached significant or extremely significant levels were usually closely related to similar habitat requirements among species, such as *Q. aquifolioides* and *Pinus densata* Mast, both of which prefer habitats on sunny slopes, grow on brown forest soil [24], have ecological adaptability to drought and barren conditions, and coexist as co-dominant species in mixed communities. Given the large number of pairwise comparisons performed and the absence of a formal adjustment for multiple testing, some statistically significant results may reflect the cumulative effect of Type I errors. Therefore, these pairwise findings should be interpreted as exploratory and hypothesis-generating rather than definitive, and they require confirmation in future studies with appropriate multiplicity control. In addition, some contingency tables may have contained relatively low expected cell frequencies, which may have affected the robustness of χ^2^-based inference despite the use of Yates’ continuity correction.

Interspecific association in forest communities is closely related to community stability [25,26]. It is generally considered that when the overall interspecific association shows a significantly positive correlation, the community has higher stability [27,28]. The M. Godron method can relatively comprehensively and systematically reflect the status of community stability, thereby providing a clearer basis for interpreting the results of interspecific association analysis [29]. Li et al. [25] used the M. Godron stability measurement method to study the tree layer of *Picea purpurea* Mast. communities and showed that the community is currently in an unstable state; Zhang et al. [30] applied the M. Godron method to forest ecosystems in Ningxia and found that the stability of *Picea crassifolia* Kom. communities were higher than that of *Larix gmelinii* var. *principis-rupprechtii* (Mayr) Pilg. communities.

The results of this study showed that the intersection coordinate of the regression model for the tree layer was (34.26, 65.74), and the Euclidean distance from the theoretical stability point was 20.17; the intersection coordinate of the shrub layer was (39.78, 60.21), and the Euclidean distance from the theoretical stability point was 27.98. This pattern reflects the ecological mechanism of the vertical structure of high-altitude montane forests. Specifically, the tree layer had higher overall stability due to its stronger cold tolerance and relatively balanced resource utilization strategy; whereas the shrub layer, as a transitional zone, was more susceptible to disturbance in its structure and function, thus showing lower stability. In this study, the stability of the tree layer in *B. platyphylla* forests was higher than that of the shrub layer, and the woody plant community may not yet represent a highly stable state. However, its successional status would require confirmation through long-term monitoring or chronosequence studies. This is consistent with the conclusions of Zhang et al. [31] on *Pinus armandii* Franch. communities and Lu et al. [32] on *Phoebe sheareri* (Hemsl.) Gamble communities.

## 4. Materials and Methods

### 4.1. Overview of the Study Area

The study area is located in the Niyang River Basin in the southeastern Qinghai–Tibet Plateau (E 92°10′–94°35′, N 29°30′–30°30′), which is the second largest tributary of the Yarlung Zangbo River. The elevation ranges from 2935 m to 5030 m, exhibiting a distinct alpine canyon landform. The terrain gradually decreases from west to east, with obvious differentiation of vegetation zones and rich biodiversity, and soils are primarily brown soils [33]. The region is jointly influenced by warm and humid airflows from the Indian Ocean and cold air masses from the north, forming a typical plateau temperate monsoon climate. The annual mean temperature is about 8.5 °C, the annual precipitation is 842 mm, the annual runoff is mainly concentrated from June to September, and the evaporation can reach 1766 mm [34,35].

### 4.2. Field Investigation and Plot Establishment

**Figure 5:**
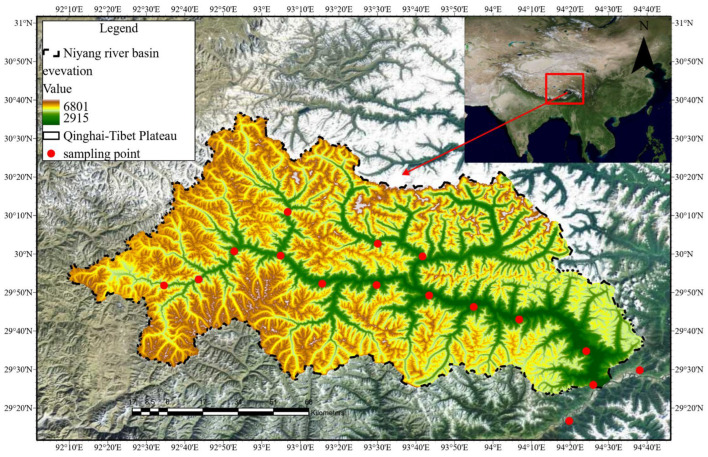
Distribution of Sampling Sites of *B. platyphylla* Forests in the Niyang River Basin.

The *B. platyphylla* forests in the Niyang River Basin belong to the temperate deciduous broad-leaved forest vegetation type. From July to August 2024, 16 representative sampling sites were established along the elevational gradient within the natural distribution range of *B. platyphylla* in the basin, from 2990 to 4100 m a.s.l. At each site, three 10 m × 10 m quadrats were randomly established, with a spacing of more than 50 m between quadrats, resulting in a total of 48 *B. platyphylla* forest quadrats (Figure 5). Tree- and shrub-layer woody plants were surveyed within the same 10 m × 10 m quadrats, and no separate shrub subplots were established. This sampling scale was used to allow direct comparison between tree- and shrub-layer community characteristics.

For each quadrat, species composition, number of individuals, mean height, and mean coverage of woody plants were recorded in detail. A Garmin eTrex handheld GPS unit was used to determine the geographic coordinates of each quadrat, and elevation, longitude, and latitude were recorded. Because the three quadrats were nested within the same site, complete statistical independence among quadrats cannot be assumed. Spatial autocorrelation and potential pseudoreplication were not formally tested; therefore, the results should be interpreted primarily as descriptive patterns of woody plant community variation along the elevational gradient.

### 4.3. Data Statistics and Analysis

#### 4.3.1. Species Importance Value

Species importance value (Important value, IV), as a comprehensive indicator for measuring its role and status in a community [36], was calculated in this study as the mean of relative abundance (relative abundance, *Ra*), relative height (relative height, *Rh*), and relative cover (relative cover, *Rc*) [37]. The calculation formula is as follows:

(1) $$IV=\frac{Ra+Rh+Rc}{3}$$

*Ra* = (number of plants of a particular species)/(total number of plants of all species) × 100%;

*Rh* = (sum of the heights of all individuals of a particular species)/(total height of all species) × 100%;

*Rc* = (sum of coverage of all individuals of a particular species)/(total coverage of all species) × 100%.

Relative abundance, relative height, and relative cover were first calculated for each species within each 10 m × 10 m quadrat. Species importance values were then calculated at the quadrat level and averaged across the 48 quadrats. Tree and shrub layers were calculated and analyzed separately.

#### 4.3.2. Niche Breadth and Overlap

The Levins index (*B*_L_) and Shannon index (*B*_S_) were used to quantify the niche breadth of the main dominant species [38]. The formulas are as follows:

(2) $$B_{L}=\frac{1}{\sum_{j=1}^{r}{P_{ij}}^{2}}$$

(3) $$B_{S}=-\sum_{j=1}^{r}\left(P_{ij}lnP_{ij}\right)$$

The Pianka niche overlap index (*O*_ik_) was used to quantify and evaluate the degree of overlap among species [39]. The formula is as follows:

(4) $$O_{ik}=\sum_{j=1}^{r}\;P_{ij}P_{kj}/\sqrt{\sum_{j=1}^{r}\;P_{ij}^{2}\sum_{j=1}^{r}\;P_{kj}^{2}}$$

The Schoener niche similarity index (*C*_ik_) was used to evaluate the degree of niche similarity [40]. The formula is as follows:

(5) $$C_{ik}=1-\frac{1}{2}\sum_{j=1}^{r}|P_{ij}-P_{kj}|$$

In the formula, *P_ij_* and *P_kj_* represent the proportions of the total importance values of species *i* and *k* in quadrat *j*. *r* is the total number of quadrats. Species absences in a given quadrat were retained as zero values in the calculations.

#### 4.3.3. Overall Association Test

The variance ratio method (VR) proposed by Schluter [41] was used to calculate the overall association among species in the community. Under the assumption of independence, when VR > 1, it indicates that the overall interspecific association is positive; otherwise, it is negative; VR = 1 indicates no association among species. The statistic W was used to determine whether a significant association exists, and W represents the degree to which VR deviates from 1.

(6) $$VR=\frac{\frac{1}{N}\sum_{j=1}^{N}{\left(T_{j}-t\right)}^{2}}{\sum_{i=1}^{S}P_{i}\left(1-P_{i}\right)}$$

(7) W=VR×N

In the formula, *T_j_* represents the total number of species occurring in quadrat *j*; *P_i_* represents the occurrence frequency of species *i*; *t* represents the average number of species per quadrat; *N* represents the total number of quadrats; and *S* represents the total number of species across all quadrats.

#### 4.3.4. Interspecific Association Test

The chi-square test (χ^2^), association coefficient (AC), and the Spearman rank correlation were used to analyze the interspecific associations of dominant species. Pairwise comparisons were conducted as exploratory analyses and, therefore, were not adjusted for multiple testing.

(1) χ^2^ test value:

To analyze interspecific association, a 2 × 2 contingency table was constructed based on the presence or absence of species in quadrats, and the χ^2^ statistic was used to analyze interspecific association. The χ^2^ value was calculated using the Yates continuity correction formula [42]. Since the χ^2^ value has no negative values, it was combined with V to determine the positive or negative interspecific association. The formulas are as follows:

(8) $$\chi^{2}=\frac{N{\left[\left|ad-bc\right|-0.5N\right]}^{2}}{\left(a+b\right)\left(c+d\right)\left(a+c\right)\left(b+d\right)}$$

(9) $$V=\frac{\left(a+d\right)-\left(b+c\right)}{\left(a+b+c+d\right)}$$

In the formula, when χ^2^ > 6.635, it indicates that the interspecific association is extremely significant (*p* < 0.01); when 3.841 < χ^2^ < 6.635, it indicates that the interspecific association is significant (0.01 ≤ *p* ≤ 0.05); when χ^2^ < 3.841, it indicates that the interspecific association is not significant (*p* > 0.05). When V > 0, it indicates that the interspecific association is positive, and when V < 0, it indicates that the interspecific association is negative.

(2) The formula of the interspecific association coefficient (AC) is as follows [43]:

(10) $$Ac=\frac{\left(ad-bc\right)}{\left(a+b\right)\left(b+d\right)}\left(ad\geq bc\right)$$

(11) $$Ac=\frac{\left(ad-bc\right)}{\left(a+d\right)\left(a+c\right)}\left(ad<bc,d\geq a\right)$$

(12) $$Ac=\frac{\left(ad-bc\right)}{\left(d+b\right)\left(d+c\right)}\left(ad<bc,d<a\right)$$

(3) The Spearman rank correlation analysis method is as follows [44]:

(13) $$r_{s}\left(i,j\right)=1-\frac{6\sum_{k=1}^{N}{\left(x_{ik}-x_{jk}\right)}^{2}}{N^{3}-N}$$

In the formula, the value range of *r_s_* is [−1,1], *x_ik_* and *x_jk_* represent the ranks of species *i* and *j* in quadrat *k*, respectively, and *N* represents the total number of quadrats. A positive *r_s_* value indicates a positive correlation, while a negative value indicates a negative correlation.

The above calculations and plotting were performed in R 4.3.2 using the spaa package version 0.2.2 [45].

#### 4.3.5. Community Stability Analysis Method

In this study, the M. Godron method improved by Zheng Yuanrun [46] was used to evaluate community stability. The specific analytical steps are as follows: (1) plant species in the community were ranked in descending order according to relative frequency, and the cumulative reciprocal percentage of species was taken as the independent variable (X), while the corresponding cumulative relative frequency was taken as the dependent variable (Y), to construct a scatter plot; (2) the scatter plot was fitted with a smoothing curve (y = ax^2^ + bx + c) to obtain a binomial equation; (3) the intersection point between this equation and the straight line Y = 100 − X was calculated. The theoretical stability point (20, 80) represents the ideal stable community state in the M. Godron method. The Euclidean distance between the intersection point and this theoretical point reflects the deviation of the observed community trajectory, with smaller distances indicating higher community stability.

## 5. Conclusions

This study investigated the niche characteristics, interspecific associations, and structural stability of dominant woody plant species in *B*. *platyphylla* forests in the Niyang River Basin of the southeastern Qinghai–Tibet Plateau. The results showed that dominant species differed in niche breadth, most species pairs had relatively low niche overlap, and interspecific associations were generally weak. The tree layer exhibited higher structural stability than the shrub layer, indicating differences in community organization between vegetation layers. These findings provide preliminary evidence for understanding the structure and coexistence patterns of dominant woody species in *B. platyphylla* forests. It should be noted that the data were collected at a single time point, lacking long-term temporal observations. Future studies should incorporate environmental gradients, particularly soil properties and hydrothermal conditions, combined with long-term monitoring of species composition and population dynamics, to further elucidate the mechanisms underlying community assembly, succession, and stability in *B. platyphylla* forests.

## Figures and Tables

**Table 1:** Importance Values and Niche Breadths of Dominant Species in the tree layer.

Abbreviation (Abbr)	Species Name	Family Name	Important Value/%	Niche Breadth	
				*B*_L_	*B*_S_
BP	*Betula platyphylla*	Betulaceae	27.43	37.16	3.71
QA	*Quercus aquifolioides*	Fagaceae	11.42	16.57	3.00
SD	*Salix daltoniana*	Salicaceae	3.24	8.72	2.41
PR	*Populus rotundifolia* var. *duclouxiana*	Salicaceae	2.85	6.86	2.13
PL	*Picea likiangensis* var. *linzhiensis*	Pinaceae	2.28	9.21	2.42
PD	*Pinus densata*	Pinaceae	1.54	5.63	2.05
SR	*Sorbus rehderiana*	Rosaceae	1.00	13.09	2.64
BU	*Betula utilis*	Betulaceae	0.59	3.33	1.28
AG	*Abies georgei*	Pinaceae	0.59	5.00	1.61
CH	*Cornus hemsleyi*	Cornaceae	0.46	5.67	1.82
LG	*Larix griffithii*	Pinaceae	0.42	2.57	1.01
PM	*Populus mainlingensis*	Salicaceae	0.30	2.00	0.69

**Table 2:** Importance Values and Niche Breadths of Dominant Species in the shrub layer.

Abbreviation (Abbr)	Species Name	Family Name	Important Value/%	Niche Breadth	
				*B*_L_	*B*_S_
RB	*Rhododendron bulu*	Ericaceae	5.18	11.13	2.70
BG	*Berberis gyalaica*	Berberidaceae	4.02	22.50	3.26
LT	*Lonicera tangutica*	Caprifoliaceae	3.53	17.63	3.14
CS	*Cotoneaster submultiflorus*	Rosaceae	3.18	11.82	2.76
DF	*Dasiphora fruticosa*	Rosaceae	2.57	7.35	2.38
LV	*Lyonia villosa*	Ericaceae	2.51	11.57	2.55
RT	*Rhododendron triflorum*	Ericaceae	2.23	9.57	2.47
RV	*Rhododendron vellereum*	Ericaceae	1.87	3.20	1.48
CA	*Cotoneaster acutifolius*	Rosaceae	1.70	10.52	2.50
RS	*Rosa sericea*	Rosaceae	1.69	9.92	2.49
RL	*Rhododendron lulangense*	Ericaceae	1.41	3.75	1.35
CB	*Caragana bicolor*	Fabaceae	1.20	3.96	1.59
RM	*Rosa macrophylla* var. *glandulifera*	Rosaceae	1.12	9.03	2.32
LF	*Leptodermis forrestii*	Rubiaceae	1.06	7.84	2.22
LH	*Lonicera hispida*	Caprifoliaceae	0.98	4.70	1.70

**Table 3:** Niche Similarity (*C*_ik_) and Niche Overlap (*O*_ik_) of Dominant Species in the tree layer.

Abbr	QA	PR	PD	SR	BP	LG	PL	AG	CH	BU	SD	PM
QA		0.36	0.20	0.48	0.36	0.24	0.27	0.33	0.20	0.00	0.06	0.12
PR	0.23		0.05	0.24	0.23	0.00	0.18	0.08	0.25	0.04	0.11	0.00
PD	0.25	0.05		0.47	0.21	0.13	0.27	0.13	0.40	0.00	0.10	0.04
SR	0.40	0.19	0.39		0.22	0.32	0.38	0.34	0.27	0.11	0.15	0.00
BP	0.33	0.17	0.16	0.16		0.08	0.33	0.14	0.20	0.29	0.46	0.18
LG	0.11	0.00	0.10	0.17	0.02		0.12	0.12	0.00	0.00	0.03	0.38
PL	0.22	0.17	0.30	0.31	0.27	0.09		0.58	0.19	0.05	0.03	0.18
AG	0.18	0.07	0.13	0.21	0.05	0.17	0.43		0.25	0.00	0.00	0.00
CH	0.12	0.27	0.34	0.24	0.09	0.00	0.23	0.20		0.05	0.06	0.00
BU	0.00	0.09	0.00	0.08	0.09	0.00	0.09	0.00	0.10		0.31	0.00
SD	0.12	0.10	0.05	0.14	0.34	0.02	0.06	0.00	0.07	0.17		0.04
PM	0.04	0.00	0.03	0.00	0.04	0.33	0.09	0.00	0.00	0.00	0.02	

Note: The letters in the table are species abbreviations, the same as in Table 1. The lower left part of the table represents niche similarity (*C*_ik_), and the upper right part represents niche overlap (*O*_ik_). The same below.

**Table 4:** Niche Similarity (*C*_ik_) and Niche Overlap (*O*_ik_) of Dominant Species in the shrub layer.

Abbr	LV	RT	RB	RS	CS	RM	LT	BG	LF	DF	CB	RV	CA	RL	LH
LV		0.47	0.10	0.25	0.58	0.36	0.30	0.47	0.16	0.03	0.03	0.01	0.40	0.32	0.02
RT	0.50		0.02	0.13	0.21	0.50	0.19	0.40	0.63	0.02	0.00	0.02	0.25	0.40	0.01
RB	0.10	0.04		0.18	0.29	0.01	0.26	0.39	0.12	0.18	0.05	0.37	0.11	0.02	0.06
RS	0.24	0.19	0.19		0.43	0.18	0.20	0.46	0.38	0.08	0.29	0.00	0.02	0.03	0.00
CS	0.38	0.21	0.29	0.38		0.26	0.32	0.49	0.23	0.07	0.10	0.25	0.08	0.15	0.00
RM	0.36	0.47	0.01	0.20	0.23		0.27	0.57	0.33	0.00	0.02	0.02	0.40	0.21	0.05
LT	0.27	0.27	0.26	0.22	0.35	0.22		0.42	0.06	0.11	0.15	0.14	0.29	0.17	0.22
BG	0.37	0.35	0.29	0.43	0.40	0.37	0.40		0.27	0.07	0.47	0.09	0.33	0.30	0.03
LF	0.19	0.48	0.14	0.35	0.21	0.31	0.06	0.20		0.08	0.00	0.04	0.12	0.03	0.00
DF	0.04	0.03	0.27	0.09	0.15	0.00	0.18	0.14	0.11		0.01	0.05	0.04	0.03	0.73
CB	0.04	0.00	0.09	0.24	0.11	0.03	0.12	0.27	0.02	0.03		0.04	0.04	0.00	0.00
RV	0.02	0.06	0.25	0.00	0.19	0.05	0.17	0.10	0.05	0.11	0.06		0.01	0.00	0.03
CA	0.39	0.25	0.10	0.05	0.09	0.32	0.26	0.23	0.11	0.06	0.02	0.03		0.18	0.06
RL	0.22	0.27	0.01	0.02	0.08	0.13	0.10	0.13	0.02	0.02	0.00	0.00	0.11		0.00
LH	0.04	0.04	0.06	0.00	0.00	0.04	0.13	0.05	0.00	0.56	0.00	0.03	0.07	0.00	

Note: The letters in the table are species abbreviations, the same as in Table 2. The lower left part of the table represents niche similarity (*C*_ik_), and the upper right part represents niche overlap (*O*_ik_). The same below.

**Table 5:** Overall Association Analysis of Dominant Species in the tree layer and shrub layer.

Different Layers	Variance Ratio	Test Statistic	χ^2^ Critical Value (0.95, 48)	χ^2^ Critical Value (0.05, 48)	Test Result
tree layer	1.85	89.04	65.17	33.10	Significantly positive association
shrub layer	1.27	60.90	65.17	33.10	Non-significantly positive association

## Data Availability

The datasets used during the current study are available on reasonable request.
